# Advanced Ceramics from Preceramic Polymers Modified at the Nano-Scale: A Review

**DOI:** 10.3390/ma7031927

**Published:** 2014-03-06

**Authors:** Enrico Bernardo, Laura Fiocco, Giulio Parcianello, Enrico Storti, Paolo Colombo

**Affiliations:** 1 Department of Industrial Engineering, University of Padova, Via Marzolo 9, Padova 35131, Italy; E-Mails: laurafiocco@hotmail.com (L.F.); paolo.colombo@unipd.it (P.C.); 2 EMPA—Swiss Federal Laboratories for Materials Science and Technology, Dübendorf 8600, Switzerland; E-Mail: giulio.parcianello@gmail.com; 3 Institut für Keramik, Glas- und Baustofftechnik, TU Bergakademie Freiberg, Agricolastraße 17, Freiberg 09596, Germany; E-Mail: enstorti@gmail.com; 4 Department of Materials Science and Engineering, The Pennsylvania State University, University Park, PA 16801, USA

**Keywords:** precursors-organic, polymer-derived ceramics, nanocomposites, silicates, SiAlON

## Abstract

Preceramic polymers, *i.e.*, polymers that are converted into ceramics upon heat treatment, have been successfully used for almost 40 years to give advanced ceramics, especially belonging to the ternary SiCO and SiCN systems or to the quaternary SiBCN system. One of their main advantages is the possibility of combining the shaping and synthesis of ceramics: components can be shaped at the precursor stage by conventional plastic-forming techniques, such as spinning, blowing, injection molding, warm pressing and resin transfer molding, and then converted into ceramics by treatments typically above 800 °C. The extension of the approach to a wider range of ceramic compositions and applications, both structural and thermo-structural (refractory components, thermal barrier coatings) or functional (bioactive ceramics, luminescent materials), mainly relies on modifications of the polymers at the nano-scale, *i.e.*, on the introduction of nano-sized fillers and/or chemical additives, leading to nano-structured ceramic components upon thermal conversion. Fillers and additives may react with the main ceramic residue of the polymer, leading to ceramics of significant engineering interest (such as silicates and SiAlONs), or cause the formation of secondary phases, significantly affecting the functionalities of the polymer-derived matrix.

## 1. Introduction

Preceramic polymers, especially in the form of organo-silicon compounds (e.g., polymers based on a backbone of Si atoms containing also C, O, N, B and H atoms), have been widely recognized for the last 40 years as an extremely powerful tool for the production of advanced ceramics. Their key advantage over conventional (powder) synthesis procedures is represented by the possibility of adopting plastic-forming techniques (e.g., fiber spinning, foaming, warm pressing, extrusion, injection molding or resin transfer molding) to generate shaped components, later transformed into the desired ceramic parts (usually defined as polymer-derived ceramics or PDCs) by thermal treatment (pyrolysis) above ~800 °C, typically in a non-oxidative atmosphere (nitrogen or argon) [1–3].

The ceramics obtained from polymeric precursors usually feature a chemical composition not achievable by other techniques: as an example, silicones (polymers with a -Si-O- backbone) yield, upon firing in a non-oxidative atmosphere, an amorphous SiCO (silicon oxycarbide) residue, which can be considered as a silica glass modified by the presence of nano-sized domains based on SiC (Si atoms surrounded by four bridging carbon atoms instead of two oxygen atoms), together with Si atoms bonded to a varying number of C atoms and C clusters (“free carbon”) [4,5]. Such nano-domains transform into distinct phases upon treatment above 1200 °C. A similar microstructural evolution is found for another well investigated class of preceramic polymers, *i.e.*, polysilazanes, yielding a SiCN (silicon carbonitride) ceramic containing nano-sized N- and C-rich areas at low pyrolysis temperature and Si_3_N_4_ and SiC nano-sized regions after heating at high temperature. Several efforts have been devoted to the control and understanding of the phase separation occurring in the ceramic residue with increasing pyrolysis temperature, with consequent (partial) crystallization, as well as to precisely describe the microstructure of the PDCs at various stages during pyrolysis [1,2,6].

A distinctive drawback of PDC technology is the poor control of shrinkage and structural integrity of the products of the polymer-to-ceramic transformation. The transformation implies the elimination of the organic moieties typical of a polymer (e.g., methyl or phenyl groups attached to the Si atoms), with consequent significant gas release (in the form of methane, benzene and hydrogen) and shrinkage (the density goes from ~0.8–1.2 g/cm^3^, typical values for a polymer, to ~2.2 g/cm^3^, a standard value for amorphous Si-based ceramics) [1,7]. Gas release not only leads to the formation of unwanted/uncontrolled porosity, but also causes a substantial cracking of monolithic pieces [7,8]. Thin-walled components (e.g., fibers, microtubes or highly porous open-celled foams) represent a notable exception, due to the intrinsic short diffusion paths for the generated gases, leading to very limited internal pressure build-up [9].

Hot-pressing of pre-pyrolyzed material has been proposed as a solution for crack-free polymer-derived ceramics since the early applications of the technology [4], and recently, spark plasma sintering (SPS) has been successfully used to produce fully dense, nano-structured components [10–12]. Although successful, a (more or less sophisticated) hot pressing treatment needs to be employed on PDC powders at least already partially pyrolyzed, as the generation of decomposition gases during pressing would create problems for the equipment.

The pioneering work of Greil [7–9,13–16] provided a fundamental solution for obtaining crack-free, almost dense (porosity typically below 15 vol%), strong monoliths with a single ceramization step, based on a more or less pronounced modification of the chemistry of PDCs. He demonstrated, in particular, the impact of two types of solid additives, or fillers:

- Inert, or passive, fillers, which are ceramic powders that do not react with the ceramic residue from the preceramic polymer, the decomposition gases or the heating atmosphere [17]. Such fillers simply dilute the preceramic polymer, therefore decreasing the amount of gas generated and the associated volume shrinkage, reducing the likelihood of forming macroscopic cracks during processing. The final ceramic has a modified chemistry in the sense that a polymer-derived matrix is accompanied by secondary phases;
- Active fillers, *i.e.*, metallic or intermetallic powders that react, during pyrolysis, with the decomposition gases generated during heating, or the heating atmosphere or (less frequently) with the ceramic residue from the preceramic polymer [7,18]. The fillers are normally quite coarse, in the (several) micron range, for handling and safety reasons, as very small metallic particles may exhibit pyrophoricity. Typical products of the chemical reactions are carbides, nitrides or silicide phases, with a significant impact on the overall shrinkage. In fact, the metal-to-ceramic transformation generally occurs with a large volume expansion, due to a large density decrease, which compensates for the shrinkage associated with the ceramic conversion of polymers. Solid particles and the *in situ* reaction with the filler reduce the amount of gas generated and the local gas pressure in the part, respectively, therefore enabling the fabrication of near-net shape, bulk, uncracked ceramic components [7].

Even when large amounts (several tens of a percent) of fillers are introduced, the viscous flow of preceramic polymers is always sufficiently high to enable the use of the abovementioned plastic forming technologies. Moreover, the use of these fillers not only allows one to produce bulk polymer-derived ceramics, but they can also provide the possibility of manufacturing ceramics with functional properties. While metals (tungsten, niobium, molybdenum, iron alloy particles) that form carbides lead to components with high hardness and wear resistance [19–22], silicides may provide magnetic and electrical functionalities (for instance, silicone resins [23] may be easily converted into magnetic ceramics or into electrically conductive ceramics, by the addition of iron silicide or MoSi_2_ powders, respectively) [24,25]. In some cases, the addition of metal oxide particles, reduced by the carbon in the preceramic polymer during pyrolysis in an inert atmosphere, can also lead to the formation of metal clusters that afford additional functional properties, but with a limited impact on the composition of the main ceramic residue [26].

Modern nanotechnology offers a wide range of nano-sized particles, which allows for a substantial updating of the concept of preceramic polymers and fillers. In particular, oxide particles, if nano-sized, are far more interesting than micro-sized ones. Nano-sized oxide particles, contrary to micro-sized ones, have a distinctive character in that they are capable of reacting directly with the ceramic residue deriving from the heat treatment of a preceramic polymer, either in inert atmosphere [27] or, more interestingly, in air, *i.e.*, upon transformation of Si-based polymers into pure silica [28–36]. Ceramics containing carbides and nitrides in an oxycarbide matrix are replaced, as products, by advanced oxide ceramics, developed in conditions of excellent phase purity. Mullite and mullite-based ceramics, from silicone resins containing γ-Al_2_O_3_ nanoparticles [28,29,31,33,34], can be seen as the starting point of this advancement in the original concept of active fillers. Micro-sized particles are far less reactive: for instance, corundum (or Al alloy) particles were also used in order to produce mullite after firing in air, but phase pure components could be obtained only after heating at very high temperatures (>1600 °C) [37,38]. The quasi-molecular mixing of the amorphous silica deriving from the thermal decomposition of the preceramic polymer in air with the oxide nano-sized fillers, on the one hand, is the reason for the very favorable reaction kinetics measured [31]. The formation of a multitude of nuclei, on the other hand, promotes a fine-grained microstructure [39], while secondary phases, if present, are also distributed at the nano-scale.

An alternative way to polymer-derived nano-structured ceramics, mainly for functional applications, is provided by the direct chemical modification of preceramic polymers using alkoxides or other chemical precursors (liquid or gaseous). This was proposed long ago for polycarbosilane, which has been modified with Ti [40–47], Al [48,49], Zr [50–52], Fe [53], Ta [54] or B [55,56], and has also been exploited for silazanes, for the realization of silicon carbonitrides containing boron (of particular interest for their exceptional high temperature properties) [57–66], early transition metals (e.g., Ti, Zr, Hf) [67–70], other transition metals (e.g., Ni, Fe, Co, Pd), aluminum [71] or others [72]. Silicone resins have also been modified using chemical precursors for metals [73–78], although the work has been devoted mainly to the fabrication of SiCO-based nanocomposites, heat treating the samples in inert atmosphere and not in air. Boron- or aluminum-modified silicones have also been produced [79–83]. It should be noted that silicon oxycarbides modified with main group or transition metals (SiMOC) have also been synthesized via pyrolysis of sol-gel, metal-modified precursors [84–87].

The metal precursors have been added for various purposes, ranging from the crosslinking of the preceramic polymer, to the control of the crystallization of the ceramic residue, from the improvement of the high temperature stability to the affording of functional properties or to the formation of catalytic particles. From the microstructural point of view, these additions have been incorporated into the Si-based ceramic residue, forming new crystalline phases, or have modified the amorphous Si-based network of the ceramic residue or have phase-separated creating new carbide, nitride, silicide, oxide or metallic crystalline phases. In some cases, the additions have allowed the preceramic polymers to retain their plastic shaping capability, while in other cases, the increase of the degree of crosslinking prevents the viscous flow of the polymer.

Finally, we must mention that also nano-sized fillers can be inert or passive. In fact, carbon nanotubes [88–90], carbon nanofibers [91] or graphene [92] (also via graphene oxide [93]) have been recently added to preceramic polymers and acted as non-reactive fillers.

The purpose of the present paper is to give an up-to-date overview of all strategies for the modification of preceramic polymers at the nano-scale, focusing on the latest developments. We will first address the chemical modifications of polymers, then discuss passive nano-sized fillers and, finally, summarize the main findings relative to the introduction of (mainly oxide) nano-sized active fillers, which are the object of specific studies conducted by our research group. We will present some case studies, with the aim of highlighting the flexibility of the approach for the manufacturing of a wide range of advanced ceramics.

### 2. Chemical Modification of the Polymer Structure

The phase separation in the ceramic residue is a fundamental issue of polymer-derived ceramics [1–3,6,77]. Amorphous SiCO and SiCN residues from polysiloxanes and polysilazanes typically start decomposing by carbothermal reactions, causing the development of SiC and Si_3_N_4_ crystals, at temperatures above ~1250 °C. The chemical modification of the polymers by the sol-gel technique, with metal alkoxides [3], represents an excellent opportunity to extend the temperature stability of the amorphous residues. As an example, Al-containing alkoxide compounds, such as alumatrane (C_6_H_12_NO_3_Al), added to commercial silicones, led to a SiAlOC residue, stable up to 1300 °C [79]. The potential of the additives, however, does not simply rely on avoiding phase separation, but also on allowing controlled crystallization with the development of new phases. The mixing of the polymer and additive occurs at a molecular level, so that the modified polymers act as a single-phase precursors for ceramic nano-composites. Above 1300 °C, the SiAlOC residue yields nano-crystalline mullite (3Al_2_O_3_·2SiO_2_), in addition to SiC, with positive effects on the structural integrity of the polymer-derived ceramic, which could be exploited for the fabrication of ceramic micro electro-mechanical systems (MEMS) [79].

**Figure 1.**
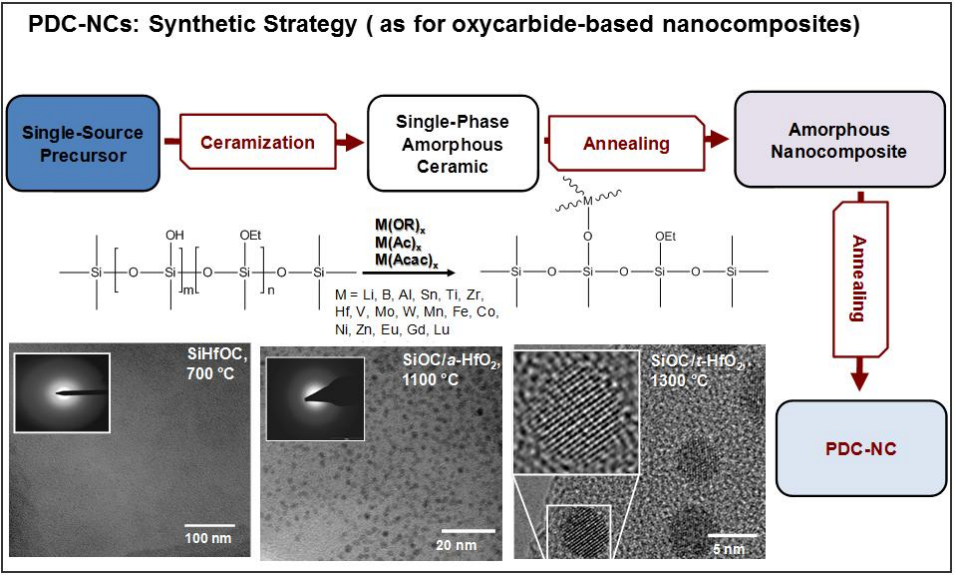
Preparative strategy for polymer-derived ceramic nanocomposites (PDC-NCs) from suitable single-source precursors (as for oxycarbide-based PDC-NCs): the TEM micrographs depict the evolution of the phase composition in the case of a SiHfOC-based material [96].

Figure 1 illustrates the typical strategy for the preparation of polymer-derived ceramic nano-composites (PDC-NCs) [73]. We can note that the process can be applied to a vast range of metal (M) alkoxides, attached to polymer chains by means of condensation reactions, involving the -OH side groups. Ionescu *et al.* [74] recently provided thermo-dynamic models useful for predicting the conversion of SiMOC residues into nano-composites; in fact, a SiCO matrix may embed metal oxides (MO*_x_*), silicides (MSi*_x_*), carbides (MC*_x_*) or metals. The stability of MO*_x_* against carbothermal reduction is essential for understanding the further evolution into silicates, on the one hand, or silicides and carbides, on the other. Lu and Mn, as an example, lead to stable oxides, in turn favoring the formation of silicate phases.

The most significant investigations that can be found in the literature concern, besides Al-alkoxides, Zr- and Hf-containing compounds. Figure 1 refers to the separation of a hafnia-rich phase, in the form of nano-sized droplets, from SiHfOC heated at 1100 °C; a further heating, at 1300 °C, leads to nano-sized tetragonal hafnia crystals (a diameter of 5 nm) [73,74]. Zr- and Hf-containing compounds are interesting, above all, for the excellent high-temperature stability of the related nano-composites [76,78,94]; zirconia and hafnia, being particularly resistant against carbothermal reduction, may form silicates, such as zircon (ZrSiO_4_) and hafnon (HfSiO_4_), stable up to 1600 °C. The same choice of additives was found to be suitable also for use at moderate temperatures (up to 250 °C), but in hydrothermal conditions. SiZrOC and SiHfOC are more resistant in this environment than pure SiCO (in turn, more resistant than SiC, being comparable to Si_3_N_4_), owing to a synergistic effect. While zirconia and hafnia have a relatively low, but appreciable, solubility in water under the testing conditions, the SiCO matrix protects the dispersed phases from the water-induced tetragonal-to-monoclinic transformation [95].

The dispersed phases, in PDC-NCs, may be interesting also for advanced applications at room temperature. As an example, Hojamberdiev *et al.* [97], exploiting the poor stability of iron oxide towards carbothermal reduction, successfully formed Fe-silicides, *i.e.*, Fe_3_Si and Fe_5_Si_3_, from a commercial silicone modified with an iron alkoxide (Fe(AcAc)_3_) after heat treatments in Ar below or above 1300 °C. Fe-silicides were developed according to a multi-step reaction, involving: (i) separation of FeO*_x_* in a SiCO matrix; (ii) reduction into Fe_3_C in SiCO; (iii) formation of a Fe-Si-C alloy; and (iv) separation of Fe_3_Si and graphitic carbon, or Fe_5_Si_3_ and β-SiC. The specific iron-containing phases led to monoliths behaving as soft magnets, which could be applied in magnetic data storage devices.

Hf-containing compounds can also be used for modifying silazanes [69,70]. In this case, the molecular structure was found to significantly affect the high-temperature behavior with respect to decomposition and crystallization phenomena. In particular, the high homogeneity achievable in polysilazane-derived ceramics favors high temperature stability, whereas hafnia-rich zones, in cyclotrisilazane-derived ceramics, promotes a pronounced decomposition.

## 3. Nano-Sized Passive Fillers

A few papers report the incorporation of carbon nanotubes, carbon fibers and graphene as nano-sized passive fillers in ceramic matrix, in order to induce or improve mechanical, thermal, electrical or rheological properties. While conventional powder-based ceramic processing has been shown to disrupt the integrity of the fillers, as well as to be not effective in their homogeneous dispersion, the polymer precursor-based approach offers several advantages for the purpose of producing ceramic matrix composites [88,90]. First, the availability of common polymer processing techniques allows one to obtain a homogeneous dispersion of a nano-sized filler in a preceramic polymer and helps to avoid the formation of agglomerates; then, the typical low pyrolysis temperatures employed can minimize the damage of the nano-structures [98]; moreover, complex shapes can be realized in small scales for microelectromechanical devices, such as MEMS [99].

Considering the brittle nature of ceramics, one of the most interesting applications of such fillers is their effect on the fracture toughness. From this point of view, Katsuda *et al.* [88] reinforced precursor-derived Si-C-N ceramics with multi-walled carbon nanotubes (MWCNTs). They demonstrated that an amount of 1–2 wt% MWCNTs produces a remarkable increase in the fracture toughness of the ceramic matrix, due to pulling-out and bridging phenomena. However, the authors highlighted that the success of this reinforcement technique is essentially linked to the nature of the nanotubes: amorphous MWCNTs were found to degrade upon thermolysis, and so, they were not effective in the toughening mechanism. Moreover, in their study, they revealed that the addition of MWCNTs up to 2 wt% does not influence the basic material properties of the matrix, such as the Young modulus, the Poisson’s ratio, the coefficient of thermal expansion and the bulk density [88].

**Figure 2.**
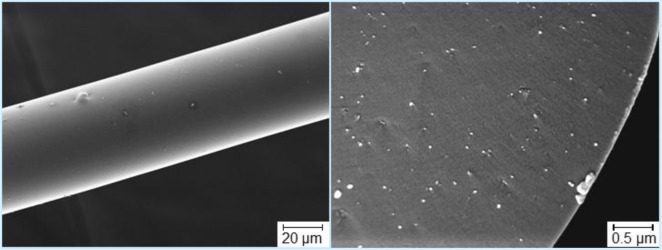
Polycarbosilazane-derived ceramic fibers with well dispersed nanotubes [100].

A further promising peculiarity of nanotubes is that their use can be suggested as a new way to tailor the rheology and the processability of spun preceramic polymer fibers, as described in the study by Kokott *et al.* [89], concerning the fabrication of SiCN-fibers by spinning the ABSE polycarbosilazane. Originally, the mentioned polymer needs thermal and catalytic treatments to increase its molecular weight in order to become suitable for the melt-spinning process. According to the authors, this can be avoided by the addition of MWCNTs: their study showed that only 1 wt% of MWCNTs can significantly improve the preceramic polymer spinnability. The key point for achieving this is producing a very good dispersion of the nanotubes: agglomerates cause a lack of stability in the spinning process and, besides, irregular fiber diameters and rough surfaces. Moreover, Kokott *et al.* pointed out that also the tensile strength of the green fibers is increased with higher fractions of MWCNTs, thus facilitating their handling [89]. An example of fiber embedding with well-dispersed MWCNTs is shown in Figure 2.

It should be noted that the achievement of a homogenous dispersion inside the ceramic matrix is the main issue concerning the use of carbon nanotubes as reinforcing fillers for polymer precursors. Such a dispersion is particularly difficult to obtain for SWCNTs, because of the strong cohesive force between individual tubes [90]. Li *et al.* investigated a novel chemical modification method involving the radical addition of an organosilicon compound to the sidewall of SWCNTs, aimed at improving the dispersion of the carbon nanotubes in a Si-C-N matrix derived from preceramic polymers. Simultaneously, the chemical modification could also be designed to provide a suitable interfacial bonding to ensure efficient load transfer from the matrix to the nanotubes [90].

Carbon-based reinforcement can also be embedded in a preceramic matrix in the shape of fibers, to enhance electrical and mechanical properties. Shibuya *et al.* [91] investigated the effect of vapor-grown carbon fibers (VGCFs) on the electrical resistivity before and after the pyrolysis of a preceramic methyl silicone resin (MSR), used as a precursor to a silicon oxycarbide ceramic (SiCO). Being that the ceramics obtained by pyrolysis were subjected to a large weight loss and shrinkage, resulting in the fracture of the bulk component during the heat treatment, sacrificial poly(methyl methacrylate) (PMMA) microbeads were incorporated to obtain microcellular VGCFs/ceramic composites, a well-established technique to eliminate cracking problems and produce highly porous components [101–103]. The bending stress and the elastic modulus of the VGCFs-reinforced ceramic were maximized when adding the PMMA microbeads in an amount of 50 wt% with respect to MSR. An optimum combination of low density, high electrical conductivity and good mechanical properties was achieved operating with 10 wt% VGCFs and 50 wt% PMMA microbeads with respect to MSR [91].

Bulk ceramics containing nano-sized reinforcements can also be fabricated from preceramic polymers when employing suitable techniques for sintering and consolidating the material, which are the crucial points for achieving dense products. For instance, graphene is a very attractive nano-sized filler, considering its unique mechanical properties that make it one of the strongest materials available, as well as its super-electrical and very good thermal properties [104–107]. This novel approach was recently followed by Rahman *et al.* [92], who fabricated graphene-reinforced silicon carbide nano-composites by incorporating graphene nano-platelets in polycarbosilane, followed by pyrolysis, grinding into fine powders and sintering by SPS. The SPS technique was necessary to consolidate the final product, thus obtaining a nano-crystalline bulk component. The mechanical properties of the reinforced samples were affected by graphene inclusions, and an increase in strength and microhardness was observed. The ceramic microstructure was also affected by the nano-platelets, with graphene inclusions found to restrict grain growth for samples processed at lower temperatures. Moreover, SiC with and without graphene inclusions showed a trend of increasing density with increasing processing temperatures, while porosity was found to decrease with increasing processing temperatures [92].

## 4. Nano-Sized Active Fillers

Table 1 summarizes the many types of silicate and oxynitride ceramics that have been so far produced by the authors, starting from preceramic polymers containing nano-sized active fillers. Preceramic polymers are typically dissolved in a suitable solvent (e.g., isopropanol or acetone), and filler powders are added, obtaining a diluted suspension (solid content 20 to 30 wt%). The mixtures are stirred magnetically for 15–20 min and then ultrasonicated for another 15–20 min in an ultrasonic bath; finally, the suspensions are dried overnight at 60 °C to produce composite (preceramic polymer + filler) powders, unless the fabrication procedure of the desired component requires the use of a fluid paste [108]. The composite powders (biphasic systems, unlike those determined by chemical modification) can then be subjected to shaping using several methods, including cold uniaxial pressing (pressure of 20 to 40 MPa), warm pressing (180 °C, 20 MPa), fused deposition, machining of warm/cold pressed blocks and coating (after dispersing the powders in water or when still in the dissolved state) [109]. The obtained shaped component is later subjected to thermal treatment to obtain the ceramic component. If the desired silicate is used in the form of powders, like in the case of inorganic phosphors for light-emitting diode (LED) devices, the thermal treatment can be applied directly to the composite powders [109]. It should be stressed that the production of silicate ceramics generally implies handling, drying and thermal treatment in air, *i.e.*, they can be prepared very easily, by conversion of the preceramic polymers into reactive amorphous silica. On the contrary, oxynitride ceramics required more stringent processing conditions. If obtained from silicone polymers, only the thermal treatment must be conducted in nitrogen in order to achieve a silicon oxycarbide (SiCO) residue, instead of silica; if obtained from polysilazanes, yielding a silicone carbonitride (SiCN) residue, drying may also be quite critical. Precautions are necessary in order to avoid the reaction of polysilazanes with H_2_O (moisture from the atmosphere or impurity in polar solvents), which may reduce the ceramic yield (by decomposition of the polymers and the consequent release of ammonia), modify the composition of the precursor and promote oxygen contaminations in the ceramic residue. Dispersions therefore need to be produced using pure apolar solvents (n-hexane, toluene) under N_2_ or Ar atmosphere (by using the Schlenk technique) and, subsequently, directly dried and cured into a tube furnace (typically at 350 °C for 1 h) under flowing N_2_. The specific processing conditions adopted for each case can be found in the published literature [110].

As previously mentioned, a general trend is that oxide fillers react with the decomposition products of the preceramic polymers, producing the desired new phases; for silicates, when developed from silicones treated in air, the reaction is particularly simple, as follows:

$$mM_{x}O_{y}+n{\mathrm{Si}O}_{\text{2 }(\text{from polymer})}\to mM_{x}O_{y}\cdot n{\mathrm{Si}O}_{\text{2}}$$ (1)

where *m*/*n* expresses the molar ratio between a metal oxide (M*_x_*O*_y_*) and silica in the desired silicate. The molar ratio is obviously associated with a weight ratio (*WR*) between oxide and silica. Given the ceramic yield (*CY*) in air of a silicone, the desired silicate will be obtained operating with a weight ratio between oxide and polymer equal to *WR*·*CY*. If the metal oxide is provided starting from a carbonate or other compounds, like sulfates, nitrates, *etc.*, the weight ratio between filler and polymer will be inferred from the *WR*·*CY* product, according to the yield in the metal oxide of the adopted compound. As an example, for wollastonite (CaO·SiO_2_), the CaO/SiO_2_ molar ratio is one, associated with *WR* = 0.93; considering the commercial silicone polymer MK (Wacker Chemie AG, see Table 1) as the silica source (a polymer with a particularly high ceramic yield; ~84% of the starting weight of the polymer is converted into silica [39], so that *CY* = 0.84), the CaO/silicone ratio will be *WR*·*CY* = 0.93 × 0.84 = 0.78; considering the yield in CaO of CaCO_3_ (0.56), we will obtain the final ratio of CaCO_3_/silicone = 0.78/0.56 = 1.40.

The small dimension of fillers allows for very favorable reaction kinetics, with the formation in most cases of fine-grained phase pure ceramics at low temperature [39], even starting from bi-phasic systems. The phase purity is particularly significant, if we consider that silicates, due to their characteristic (partially covalent) chemical bonding, generally feature a poor ionic interdiffusion [116] and no liquid phase (which could accelerate the diffusion) forms during the process of the conversion of preceramic polymers and the reaction with fillers. The limited dimensions of crystals of the desired phase favors the incorporation of secondary phases; in particular, ZrO_2_ nano-particles form zirconia agglomerates that do not exceed the critical size for tetragonal-to-monoclinic transformation upon cooling, thus posing the conditions for an effective transformation toughening (with ceramics composites achieving a fracture toughness, *K*_C_, of 6.5 MPa·m^0.5^) [34].

**Figure 3.**
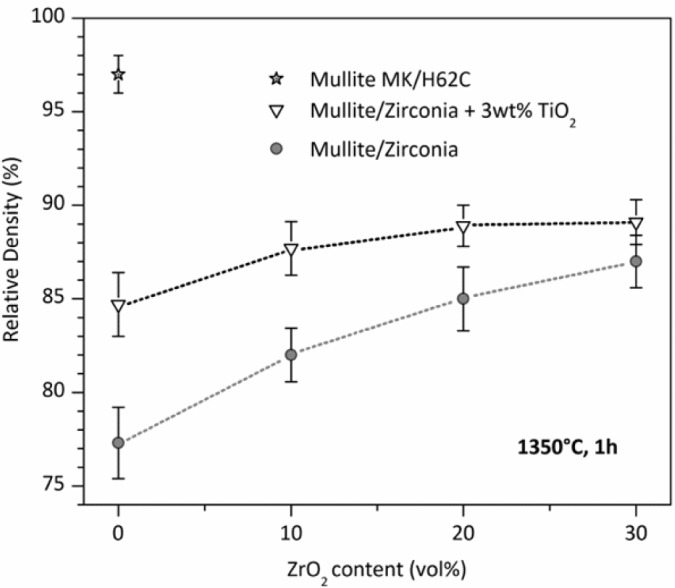
Mullite-based ceramics from MK polymer filled with γ-Al_2_O_3_ nanoparticles: the effect of secondary filler (ZrO_2_ nano-particles), sintering aid (TiO_2_ nano-particles) and partial changes in the starting polymer (50% silica provided by MK polymer; 50% provided by H62C polymer).

While phase purity and limited grain size are easy to achieved, densification remains an important issue. The poor interdiffusion, if positive for limiting the grain growth, has a negative impact on density. Dense zones are generally surrounded by a number of submicron pores, which could be ascribed to the gas evolution upon ceramic transformation, as well as incomplete sintering. The porosity in pure mullite from MK silicone and nano-sized γ-Al_2_O_3_, as shown in Figure 3, exceeds 20%. The introduction of ZrO_2_, in order to prepare zirconia-toughened mullite, is advantageous mainly for the dilution of the transforming mass; the higher is the content secondary oxide, acting as an inert filler, the lower is the amount of polymer, with lower gas evolution. For composites with 30 vol% ZrO_2_, the densification, however, is still poor.

An important improvement, especially for pure mullite, comes from the introduction of further fillers. As an example, TiO_2_ nano-particles promote the densification by decreasing the viscosity (above 1200 °C) of the amorphous silica provided by the polymer; in other words, silica is modified by the incorporation of titania, and transient viscous sintering (as reported by other authors [117,118]) may occur before the nucleation of the mullite phase. Figure 3 clearly shows that the relative density of pure mullite goes from ~77% to 85%; the effect is less and less important, with increasing ZrO_2_ addition, consistent with the reduction of silicone and, consequently, of silicone-derived silica. Improvements in the densification, associated with the presence of TiO_2_ nano-particles, were observed also for other systems, such as zircon (ZrSiO_4_) [111] and, more recently, forsterite (Mg_2_SiO_4_). The mixing of titania with silica, in forsterite, is testified by the development of a solid solution, e.g., Mg_2_Si_0.9_Ti_0.1_O_4_, at only 1100 °C [112].

**Figure 4.**
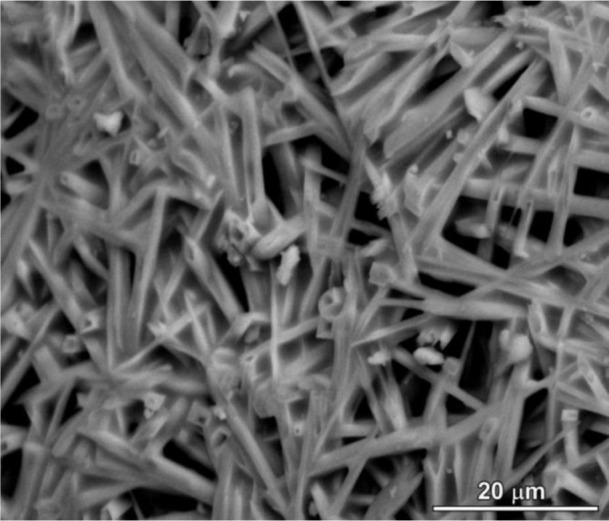
Detail of mullite ceramic with acicular microstructure, obtained from MK polymer filled with γ-Al_2_O_3_ nanoparticles and borax.

The limited interdiffusion is a constraint also for the morphology of crystals. All polymer-derived mullite-based materials developed so far possess, in fact, equiaxed grains. A remarkable exception is represented by recently prepared mullite ceramics, clearly exhibiting anisotropic crystal growth, as illustrated in Figure 4. Such morphology is due to the introduction of small amounts of borax (hydrated sodium borate, 3 wt% referring to the weight of mullite) in silicone/γ-Al_2_O_3_ mixtures. The role of B_2_O_3_ in lowering the mullitization temperatures and in promoting the anisotropic growth of mullite crystals has been widely discussed in the literature [119]; the use of a borate, in the present case, was aimed at providing a low viscosity intergranular phase (in turn, stimulating anisotropic growth [120,121]), that could be dissolved easily by acid leaching (it should be noted that B^3+^ ions may be incorporated in mullite in relatively high amounts, while Na^+^ can be accommodated in the crystal structure only in limited quantities [122,123]). Figure 4 provides an example of mullite ceramic (fired at 1300 °C, for 1 h) after acid leaching; although quite preliminary, the image demonstrates the potential for the fabrication of highly porous bodies, composed of interlocking mullite fibers. Such cellular ceramics could provide an alternative to acicular mullite ceramics prepared by much more complicated thermo-chemical processing (e.g., controlled decomposition of fluoro-topaz [124]).

The density of polymer-derived mullite may be improved also by changing the silica source. In particular, with 50% of the silica provided by MK and 50% by another polymer, H62C (a liquid polymer, with a silica yield of 58% [39]), it was possible to achieve a relative density of 97% for pure mullite, as illustrated in Figure 3. In the authors’ opinion, this could be justified by the different molecular structure of the polymers, leading to amorphous silica with a different degree of network connectivity and a number of defects; in other words, a more defective silica network would lead to Si-O network fragments that can be more easily accommodated in the mullite structure. This hypothesis has not been yet verified by dedicated experiments (such as detailed Raman spectroscopy) and will constitute the focus of future work.

Dense mullite-based ceramics, e.g., with relative density from 93% to 97%, can be produced also by another refined approach, involving changes in the filler and in the firing atmosphere. Riedel *et al.* [27] used nano-sized γ-Al_2_O_3_ powders functionalized at the surface by octylsilane groups; mixed with MK polymer, such fillers proved to be more reactive, leading to the formation of a larger weight fraction of mullite crystals at lower processing temperatures (1300 °C) as compared to un-functionalized nano-γ-Al_2_O_3_ filler. Such improved reactivity is ascribed to the enhanced homogeneity of the distribution of alumina nano-particles in the starting polysiloxane system. Since the firing treatments were performed in nitrogen, MK converted into a silicon oxycarbide (SiCO) ceramic, rather than into silica; the interaction with functionalized nano-γ-Al_2_O_3_ determined the formation of SiC crystals, in the range of 1–8 nm, embedded in a mullite matrix, with crystals in the range of 60–160 nm. The obtained crack-free monoliths may be seen as a refinement of analogous SiC/mullite nano-composites from polymethylsiloxane gels filled with α-Al_2_O_3_ (fired at higher temperatures) [125] and serve as prototypes for complex shaped high temperature- and corrosion-resistant ceramic devices. Similar results were obtained by the same research group [126], by using MK coupled with aluminum nano-sized particle filler; the different reaction paths (involving the oxidation of Al nano-particles) led to ternary composites, with nano-sized α-Al_2_O_3_ as an additional phase together with SiC and mullite.

In some cases (e.g., in zircon, forsterite and cordierite ceramics [111–113]), the partial replacement of MK with H62C proved to be advantageous for avoiding extensive cracking of samples. Several factors may justify the increase of compactness obtained when substituting part of MK by H62C; besides a different molecular structure of the polymeric precursors (in turn, providing a different ability to relax structural rearrangements and eliminate gases during the pyrolysis step, without the local pressure accumulation phenomena), the substitution could be advantageous for:

- the reduction of the gas release during polymer cross-linking, due to different cross-linking reactions occurring in the two polymers;
- the application of a crosslinking step (30 min at 250 °C) before powder compaction, which involves a certain degree of shrinkage, which is consequently absent in the subsequent pyrolysis.

Binary systems, comprising silica and one metal oxide, may be complicated by the formation of multiple silicate phases, each with a specific molar balance between silica and the metal oxide. A fundamental example is given by silica coupled with CaO, leading to many silicates with a different CaO/SiO_2_ molar ratio, such as CaO·SiO_2_ (=CaSiO_3_), 3CaO·2SiO_2_, 2CaO·SiO_2_ and 3CaO·SiO_2_, in their polymorphic variants, useful mostly for applications in the field of biomaterials [32,35]. Local concentrations of CaO, released from a filler, such as CaCO_3_, may lead to the development of a silicate with a higher CaO/SiO_2_ ratio than the desired one; as an example, micro-sized CaCO_3_, in a filler/silicone formulation theoretically yielding wollastonite (CaO·SiO_2_), was found to favor the formation of di-calcium silicate (2CaO·SiO_2_), whereas nano-sized CaCO_3_, in the same mixing conditions, led to almost pure wollastonite [35]. The different molecular structure of the polymeric precursors, in the case of calcium silicates, was found to affect also the polymorphism: the use of H62C instead of MK led to traces of additional phases (including α-phase or “pseudowollastonite”), in addition to the β-phase, normally obtained at the adopted processing temperature (900–1100 °C [32,35]). Ring-structured silicate variants (such as the α-phase) [127], instead of chain-structured ones (such as the β-phase), might be produced, because of the availability of short Si-O fragments, enhanced by the use of a polymer with a lower molecular weight, such as H62C.

Other complex binary systems correspond to the interaction of silicones with MgO and Y_2_O_3_. Enstatite contaminations (MgO·SiO_2_ = MgSiO_3_) can be produced in a formulation theoretically yielding forsterite (2MgO·SiO_2_ = Mg_2_SiO_4_), to be used as a biomaterial [128] and a high-performance dielectric [129]. Silicones filled with nano-sized MgO are highly reactive, since forsterite can be obtained already at 800 °C, but the characteristic poor ionic interdiffusion in silicates impedes the complete dissolution of MgO, with consequent development of enstatite in MgO-poor zones. Nano-sized titania, forming a solid solution (Mg_2_Si_0.9_Ti_0.1_O_4_), besides providing an improvement in the densification, as previously mentioned, is fundamental in removing both un-reacted MgO and enstatite contaminations [112].

Yttria and silica can combine into two silicates, *i.e.*, they form mono- (Y_2_O_3_·SiO_2_ = Y_2_SiO_5_, or Y-MS) and di-silicates (Y_2_O_3_·2SiO_2_ = Y_2_Si_2_O_7_, or Y-DS (yttrium di-silicate)) [109]. Y-MS is practically monophasic, being subjected only to a dislocative (*i.e*., martensitic) transformation between the X1 phase (low temperature phase) and the X2 phase (high temperature phase) [130]. Y-DS, on the other hand, features many polymorphs (y, α, β, γ, δ, z) [131,132]; all the forms are stable in a certain temperature range, but high temperature polymorphs can be retained at room temperature, due to extremely slow phase transformations [130]. A particular polymorph may be associated with a specific processing procedure [132]: for instance, whereas sol-gel processing favors the formation of the α-phase [133,134], hydrothermal synthesis is known to promote the development of the y-phase [135]. Using MK silicone containing Y_2_O_3_ nano-powders [109], both silicates can be obtained at particularly low temperatures (1000–1350 °C), in analogy with the results of the more complicated sol-gel processing; Y-DS is obtained in its most stable variant (γ-phase). Contaminations of Y-MS, in Y-DS, are quite difficult to remove, but they do not represent an issue, especially for some applications; in fact, Y-DS is applied (due to the similarity in the coefficient of thermal expansion) in anti-oxidation coatings on SiC ceramics (see below), and Y-MS may combine with SiO_2_, from the partial oxidation of SiC, to yield additional Y-DS [136].

Ternary systems pose difficulties, besides the possible formation of many silicate phases, according to multiple combinations among silica and oxides (e.g., the CaO-MgO-SiO_2_ system may lead to CaMgSi_2_O_6_, known as diopside, or Ca_2_MgSi_2_O_7_, known as akermanite, and so on), also for the possible formation of silica-free compounds. As an example, cordierite (2MgO·2Al_2_O_3_·5SiO_2_, or Mg_2_Al_4_Si_5_O_18_) and gehlenite (2CaO·Al_2_O_3_·SiO_2_, or Ca_2_Al_2_SiO_7_) may be accompanied by Mg and Ca aluminates, respectively. The formation of silica-free compounds impedes the complete incorporation and reaction of oxides with the polymer-derived silica, with the risk of the formation of cristobalite. The contaminations may be removed by adjusting the firing temperatures (MgAl_2_O_4_ disappears in polymer-derived cordierite fired at 1350 °C [113]) or by enhancing the ionic interdiffusion, by the formation of solid solutions (*i.e*., more “open” crystal structures). In particular, the partial replacement of Ca^2+^ ions with Eu^3+^, aimed at developing luminescent materials, with corresponding tuning of the Al/Si ratio (the formation of Ca_2−2_*_x_*Eu_2_*_x_*Al(Al_1+2_*_x_*Si_1−2_*_x_*O_7_) solid solutions), is effective in suppressing Ca-aluminate phases [114].

(Silicon) oxynitrides are intrinsically challenging to produce from preceramic polymers and fillers, due to the very complex reaction paths possible, involving carbothermal reduction and nitridation. C in SiCO or SiCN ceramic residues, deriving from polysiloxanes and polysilazanes, respectively, when treated in nitrogen, must be removed as CO. These interactions may be summarized by the following general reaction schemes:

$$\mathrm{Si}CO+M_{x}O_{y}+N_{\text{2}}\to M–\mathrm{Si}–O–N+CO↑$$ (2)

$$\mathrm{Si}CN+M_{x}O_{y}+N_{\text{2}}\to M–\text{Si}–O–N+CO↑$$ (3)

where M*_x_*O*_y_* stands for a metal oxide, inserted as a nano-sized powder. Ideally, the adoption of SiCO or SiCN would be conditioned only by the *x*/*y* ratio of the nano-sized filler and by the stoichiometry of the desired oxynitride. However, there are at least four fundamental issues to consider:

- The balance of Si, O, N and C atoms in the ceramic residues of preceramic polymers is not strictly defined; it is highly sensitive to the processing conditions, especially for polysilazanes (for which significant oxygen contaminations in the SiCN residue should be taken into account);
- Silicate formation may precede the formation of oxynitride phases; in particular, silicones and nano-sized alumina lead to mullite (3Al_2_O_3_·2SiO_2_), subsequently subjected to carbothermal reduction and nitridation, with the separation of corundum in addition to the desired SiAlON phase (3Al_2_O_3_·2SiO_2_ + 6C + 2N_2_ → Si_2_Al_4_O_4_N_4_ + 6CO + Al_2_O_3_) [30];
- Reducing conditions may lead to the formation of other by-products, e.g., SiO gas; the escape of SiO has, obviously, an impact on the stoichiometry of the obtained oxynitride;
- The ceramic residue does not react as a homogeneous mixture of Si, O, N and C atoms; e.g., a SiC separate phase may first form, with an impact on the stoichiometry of the obtained oxynitride and on secondary reactions (e.g., 2SiO_2_ + SiC → 3SiO + CO) [137,138].

The development of many gaseous species impedes the obtainment of monolithic samples. This finding, however, does not compromise the potential of the approach for the development of porous bodies, ceramic joints (e.g., the joining of pre-shaped SiAlON pieces by ceramization of the silicone/nano-alumina interlayer [36]) and powders, to be used as a sintering aid for Si_3_N_4_ or as phosphors, in the case of doping with rare-earth ions [39,110]. The contaminations may be suppressed by the reformulation of polymer/filler ratios and/or insertion of additional fillers: in particular, in β-SiAlON ceramics, the Si and N content may be adjusted by the use of amorphous Si_3_N_4_ or of polymers with an optimized yield of these elements (perhydropolysilazane) [110]. Some specific systems, finally, follow a more straightforward reaction path: for instance, we recently found that Y-Si-O-N phases can be obtained by direct reaction, *i.e.*, with no formation of silicates and the separation of SiC [39].

## 5. Products and Selected Case Studies

Table 1 reports the many ceramic systems investigated so far, with some remarks referring to the possible applications. Detailed information can be found in the cited literature, but we can list four general types of products, each associated with some applications.

### Monoliths

Mullite, zircon, cordierite and SiAlON are widely appreciated as high temperature ceramics, especially for their high thermal shock resistance (in turn, associated with the relatively low coefficient of thermal expansion); in addition, silicates, such as mullite and forsterite, are generally used in high-performance electronic packaging [39]. The proposed approach could be highly attractive for the associated shaping possibilities: in fact, simple uniaxial pressing (especially warm pressing, conducted at temperatures in the range of 150 to 180 °C) leads to the formation of components with controlled geometries, high green strength and reduced green porosity; the firing may lead to defect-free ceramic parts, with uniform shrinkage, resulting from both the decomposition of the preceramic polymer and the formation of the crystalline phases. Machining can be easily accomplished when the component is still in the green state, thereby reducing the wear of the tools, and shape retention, before ceramic conversion, is guaranteed by the presence of a network of solid particles, which limits the viscous flow of the polymer [39]. The integrity of samples, after firing, can be favored, besides the choice of polymers (e.g., partial replacement of MK with H62C, as mentioned above), by the introduction of secondary fillers, not reacting with the ceramic residue of preceramic polymers, such as TiO_2_ (in forsterite ceramics), natural zircon (zircon “seeds”, in zircon ceramics), SiC (in SiAlON ceramics) and hydroxylapatite (in Ca-based silicates, to be used as biomaterials) micron- and nano-sized powders [35,111,112,115,139].

### Cellular ceramics

Highly porous ceramics, to be used as filters or highly porous scaffolds for bone tissue engineering, may be seen as the “easiest” product from preceramic polymers [39]. While for the production of dense monoliths, several strategies (the selection of the polymer, the introduction of secondary fillers) must be applied to reduce the porosity, starting from that fundamentally provided by the gas evolution occurring upon ceramic conversion of preceramic polymers, cellular ceramics fully exploit the shaping possibilities offered by the particular raw materials. Foaming of mixtures of a preceramic polymer plus nano-sized fillers can be carried out using a variety of approaches, from direct foaming using additives that decompose at low temperature (80–350 °C) releasing a large volume of gas, to the mixing with sacrificial solid polymeric microbeads, followed by cold or warm pressing (the microbeads are eliminated during firing, leaving behind a large amount of pores of the desired size) [39]. In addition, since the preceramic polymers are soluble in a large variety of organic solvents, viscous pastes can be easily obtained and processed through syringes or, more efficiently, by automated manufacturing methods (e.g., fused deposition), leading to 3D scaffolds [108]. The macro-porosity, provided by foaming, can be coupled with micro-porosity, from ceramic conversion, thus yielding components with hierarchical porosity. It should be noted that the additives releasing gasses may be of both organic and inorganic origin; in particular, hydrated salts, like borax (as mentioned above, sodium borate decahydrate, Na_2_B_4_O_7_·10H_2_O), are interesting for a double action, *i.e.*, they provide water vapor (useful for foaming), by decomposition, and a liquid phase upon firing (in turn, favoring the interdiffusion), as recently shown for the manufacturing of akermanite (Ca_2_MgSiO_7_) ceramics [115].

### Coatings

**Figure 5.**
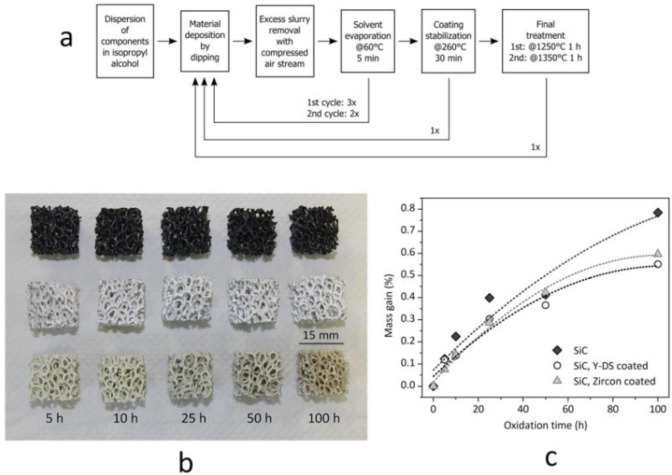
Silicate coatings on Si-SiC foams: (**a**) diagram of the procedure of coating/heat treatment; (**b**) visual appearance of samples (top line: un-coated Si-SiC foams; middle line: polymer-derived coating with Y-silicate; bottom line: polymer-derived zircon coating) (edge length: 15 mm); (**c**) weight gains with increasing oxidation time at 1200 °C.

Monolithic and cellular components often rely on the grinding of the solid residue, in turn determined by the drying of silicone-based suspensions, in large glass containers, in order to prepare “composite” powders. The grinding step can be avoided if preceramic polymers and fillers are used for coatings. Suspensions based on silicones and nano-sized fillers, as an example, were used to coat both SiC foams and SiAlON monoliths. The drying directly on the substrates determines the formation of a composite layer, later ceramized upon firing in air or nitrogen, respectively. In the first case, the coating is aimed at the development of a silicate environmental barrier coating, aimed at protecting SiC from extensive oxidation; in the second case, the coating acts as a polymeric glue, at low temperature (SiAlON pieces are joined across the coated surface), and transforms into a SiAlON joint, at high temperature [36]. Figure 5 illustrates a case study on environmental barrier coatings, concerning yttrium di-silicate (Y-DS) and zircon coating on Si-SiC foams; the formulations used in the experiments are reported in Table 2. For the realization of sufficiently thick and homogeneous depositions, an optimized multi-coating procedure was followed, as illustrated by Figure 5a. It consisted of repeated steps of deposition, solvent removal after deposition (made at 60 °C for 5 min) and coating stabilization (made at 260 °C), in order to cross-link the polymeric phase and to fix the coating on the SiC substrates. After this procedure, the final heat treatment was carried out in air with a 2 K/min heating rate. The overall procedure was repeated twice, with different maximum temperatures (1 h holding time), 1250 °C for the first cycle (aimed at the consolidation of the deposit, with no melting of Si) and 1350 °C for the second (aimed at phase development). In order to compensate for the shrinkage, secondary (inert) fillers were used in both formulations (mullite for the Y-DS coating, natural zircon for zircon coatings); kaolin clay was used as a viscosity modifier. As shown by Figure 5b, uniform coatings were formed; from SEM observations, the thickness was estimated to be ~100 μm. Although still to be improved (the coatings contain some micro-cracks), the strategy is quite promising, since the oxidation of the substrate was effectively reduced, as illustrated by the weight gains of foams heated at 1200 °C (for periods from five to 100 h), in Figure 5c.

### Powders

**Figure 6.**
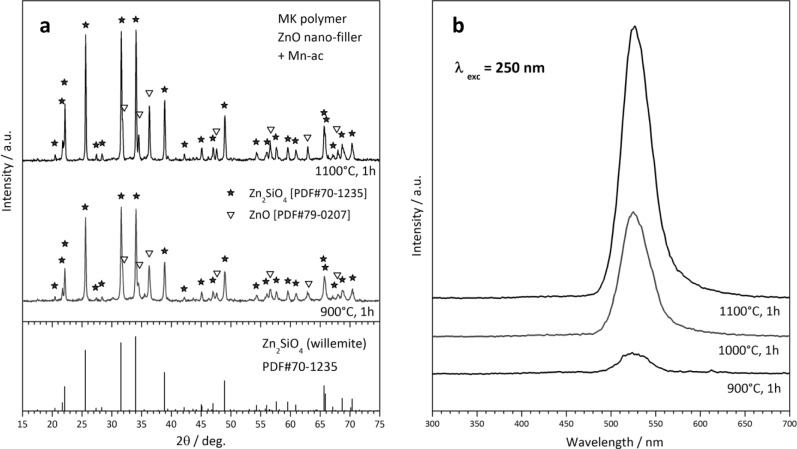
X-ray diffraction patterns (**a**) and luminescence spectra; (**b**) of polymer-derived Mn-doped zinc silicate phosphors.

A second type of “unshaped” polymer-derived product is represented by powders, from the direct firing of silicone/filler mixtures. The X-ray diffraction patterns in Figure 6a testify the easy obtainment of willemite, *i.e.*, zinc silicate (Zn_2_SiO_4_ or 2ZnO·SiO_2_), from MK polymer and ZnO nano-particles. Some peaks of unreacted ZnO remain, but we should focus on the particularly low temperature (900 °C) at which the desired phase can be detected. As reported above, “nano-filled” silicones can be actually seen as an alternative to more complicated sol-gel processing. Figure 6a actually refers to Mn-doped willemite (Zn_1.9_Mn_0.1_SiO_4_), for which we readopted a strategy already applied for wollastonite ceramics [32]. The dopant (Mn^2+^) was not provided by means of MnO nanoparticles, but starting from Mn-acetate (Mn-ac). The manganese salt was used after dissolution in distilled water (10% solid content); the acetate solution was added dropwise to a silicone solution (MK in isopropyl alcohol), embedding also ZnO nanoparticles, under magnetic stirring; a non-ionic surfactant (Pluronic P 123, BASF Corporation, Florham Park, NJ), added to the silicone solution (1 g for 80 mL solution), was useful for obtaining homogeneous emulsions. The obtained Mn-doped willemite powders, after drying and ceramization, could be effectively used as green phosphors, even in the presence of some unreacted ZnO; as illustrated by Figure 6b, samples fired at 900–1100 °C exhibit a strong luminescence peak at 520–530 nm, after excitation in the UV range (250 nm), in analogy with what was reported for willemite-based phosphors obtained by conventional techniques [140].

**Figure 7.**
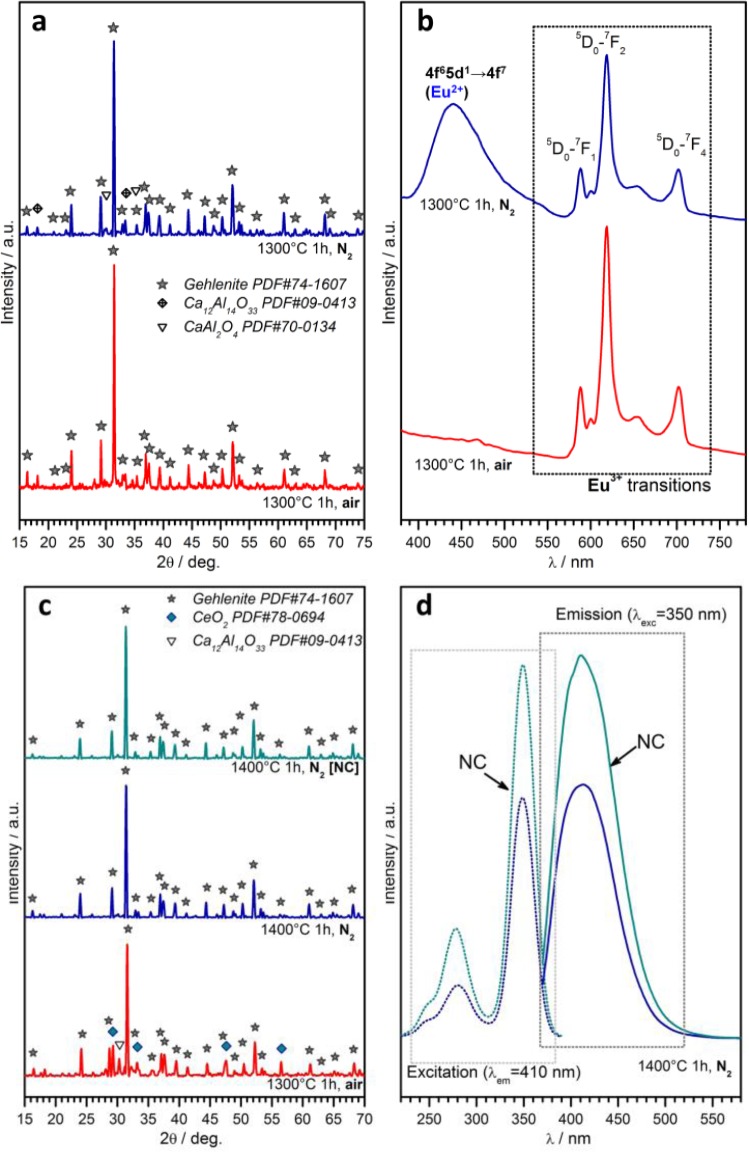
Development of polymer-derived gehlenite phosphors in air or in nitrogen atmosphere: (**a**,**b**) Eu-doped gehlenite (X-ray diffraction and luminescence); (**c**,**d**) Ce-doped gehlenite (X-ray diffraction and luminescence).

A further case study, in the field of phosphor powders, concerns gehlenite (2CaO·Al_2_O_3_·SiO_2_) ceramics. This system has an impressive “tunability”, depending on the choice of dopants, on the ratios between main oxides and on the firing atmosphere. As reported above, the partial replacement of Ca^2+^ ions with Eu^3+^ could be compensated for by the tuning of the Al/Si ratio, forming the solid solution Ca_1.86_Eu_0.14_Al(Al_1.14_Si_0.86_O_7_), which represents the only crystal phase for treatments in air (firing at 1300 °C, for 1h), as shown by Figure 7a [114]. The obtained powders, as illustrated by Figure 7b, exhibit a strong red luminescence (main peak at ~620 nm) after excitation in the UV range, as expected by a previous paper on the tested formulation [141]; it should be highlighted, however, that the previous paper referred to conventional synthesis, helped by fluxes (e.g., boric acid). In addition, tests in nitrogen were applied, starting from a silicone/nano-fillers system, with the possibility of modifying the emission characteristics, according to reduction reactions, in turn promoted by the conversion of MK polymer into SiCO instead of pure silica. As shown by Figure 7a, gehlenite remained the main crystal phase, but some CaAl_2_O_4_ formed; while gehlenite could keep Eu^3+^ ions, the secondary phase is known to embed Eu^2+^ ions [142], made available by the presence of carbon in the ceramic residue of the polymer (Eu_2_O_3_ + C → 2 EuO + CO↑), leading to the blue emission (centered at 440–450 nm) in the spectra of Figure 7b.

Polymer-derived gehlenite is interesting also when doping with Ce^3+^ instead of with Eu^3+^. For treatments in air, the reduction of CeO_2_ into Ce_2_O_3_ was not complete, even at 1300 °C, as testified by the presence of un-reacted oxide (see Figure 7c). On the contrary, gehlenite was the only crystal phase for treatments in nitrogen (1400 °C), applied to both charge-compensated and not charge-compensated (NC) formulations, *i.e.*, formulations nominally leading to Ca_1.95_Ce_0.05_Al_2.05_Si_0.95_O_7_ and Ca_1.95_Ce_0.05_Al_2_SiO_7_, respectively. After excitation in the near-UV (350 nm), powders from both formulations exhibited an intense blue-violet emission (peak at ~420 nm; see Figure 7d, reporting also the absorption spectra), consistent with the literature [143]. The more intense emission from the NC sample is attributable, in the authors’ opinion, to the possible partial nitridation, *i.e.*, replacement of Si-O bonds with Si-N bonds; in fact, nitrogen-gehlenite (Ca_2_AlSi_2_O_6_N [144]) is feasible in the presence of a low Al/Si ratio; NC could be more prone to nitridation, owing to the lower content of Al^3+^ compared to Si^4+^. Furthermore, this hypothesis has not yet been verified by dedicated experiments, but will certainly constitute the focus of future work.

### 6. Concluding Remarks

Modifications at the nano-scale of preceramic polymer enable the fabrication of a vast range of advanced ceramics to be applied in numerous engineering and specialized applications. These modifications are quite simple, since: (i) preceramic polymers are in most cases easily dissolved in organic solvents, so that nano-sized fillers, active or passive, can be dispersed homogeneously; and (ii) the backbone of a preceramic polymer generally presents side groups, such as -OH, which can easily react with metallorganic compounds, with the development of metal-modified polymers.

The versatility of the technology has already been appreciated in the fabrication of components suitable for biological, high temperature and functional (e.g., phosphors) applications, but we can say that, probably, we are just at the beginning; in fact, several improvements and refinements are expected in many aspects, including:

- Processing environment: composition and phase assemblage of non-oxide ceramics (oxycarbide, oxynitrides) from reactive systems (metal-modified polymers or polymers embedding active fillers) depend on the processing environment (flow rate of nitrogen or argon, type of furnace), so that reproducibility could be an issue; products should be associated with a specific protocol, to be carefully defined;
- Availability of preceramic polymers: from Table 1, it can be observed that only a limited number of polymers was used for many types of ceramics; it would be very interesting to test more of the various silicone resins currently available on the market, considering the changes in densification, developed phases and integrity of samples, associated with the replacement of MK with H62C (which could be seen as only the first example);
- Ceramic compositions: silicates, *i.e.*, metal oxide + silica systems, are very numerous by themselves: infinite combinations can be explored in terms of solid solutions or composites; oxycarbides and oxynitride systems have an even more pronounced variability (e.g., in SiAlON ceramics, the balance among constituents is typically conditioned by the replacement of the Si-N bond with Al-O bond processing technologies: polymers, by themselves, offer many distinct processing strategies; bi-phasic systems or metal-modified polymers may possess a lower flowability, compared to pure polymers, so that processing conditions and additives need to be carefully selected, but nano-modified preceramic polymers remain open to a vast range of forming technologies.

## Figures and Tables

**Table 1.** Summary of silicate and oxynitride ceramics from preceramic polymers and nano-sized fillers prepared at the University of Padova (* not previously published).

Ceramic phase	Polymer	Nano-sized filler	Secondary components	Remarks	Reference
Mullite (3Al_2_O_3_·2SiO_2_)	MK	γ-Al_2_O_3_ (15 nm, E)	–	Monoliths grain size <300 nm	[28,31]
	MK + H62C			Denser samples	*
	H62C		Borax	Acicular mullite crystals	*

ZTM (Zirconia Toughened Mullite)	MK	γ-Al_2_O_3_ (15 nm, E)	ZrO_2_ (13 nm, E) TiO_2_ (13 nm, E)	Reinforced monoliths (*K*_C_ ~ 6.5 MPa·m^0.5^)	[34]

Wollastonite (CaO·SiO_2_)	MK	–	Ca-acetate	Monoliths and foams	[32]
		CaO (<170 nm, D)	–		
		CaCO_3_ (90 nm, P)	n-HAp, m-HAp		[35]
	MK + H62C	CaCO_3_ (90 nm, P)	TEOS	3D scaffolds	[108]

Yttrium mono-silicate (Y_2_O_3_·SiO_2_)	MK	Y_2_O_3_ (30–50 nm, I)	Eu_2_O_3_ (45-60 nm, C)	Phosphor powders	[109]
Yttrium di-silicate (Y_2_O_3_·2SiO_2_)		–	–	Environmental barrier coatings	*

Zircon (ZrO_2_·SiO_2_)	MK, H62C	ZrO_2_ (13 nm, E)	TiO_2_ (13 nm, E) Zircon seeds	Monoliths, environmental barrier coatings	[111]

Forsterite (2MgO·SiO_2_)	MK, H62C	MgO (30 nm, I)	TiO_2_ (13 nm, E) m-TiO_2_	Monoliths for dielectric components	[112]

Willemite (2ZnO·SiO_2_)	MK	ZnO (30–50 nm, I)	Mn-acetate	Phosphor powders	*

Cordierite (2MgO·2Al_2_O_3_·5SiO_2_)	MK, H62C	γ-Al_2_O_3_ (15 nm, E) MgO (30 nm, I)	–	Monoliths and foams	[113]

Gehlenite (2CaO·Al_2_O_3_·SiO_2_)	MK	γ-Al_2_O_3_ (15 nm, E) CaCO_3_ (90 nm, P)	Eu_2_O_3_ (45-60 nm, C) CeO_2_ (20 nm, M)	Phosphors for treatment in air or in N_2_; Ce-doping effective in N_2_	[114]

Akermanite (2CaO·MgO·2SiO_2_)	MK, H62C	CaCO_3_ (90 nm, P) MgO (30 nm, I)	m-HAp Borax	Monoliths and foams	[115]

Hardystonite (2CaO·ZnO·2SiO_2_)	MK	γ-Al_2_O_3_ (15 nm, E) ZnO (30–50 nm, I)	Eu_2_O_3_ (45-60 nm, C)	Phosphor powders	*

β′-SiAlON	MK, H44	γ-Al_2_O_3_ (15 nm, E)	Si_3_N_4_, AlN, SiC	Monoliths, foams, ceramic joints	[30,33,36]
	PSZ20, NN120-20		Si_3_N_4_ (20 nm, G) Eu_2_O_3_ (45-60 nm, C)	Monoliths, phosphor powders	[39,110],*

Ca-α′-SiAlON	PSZ20	γ-Al_2_O_3_ (15 nm, E) CaCO_3_ (90 nm, P)	Eu_2_O_3_ (45-60 nm, C)	Phosphor powders	*

Y-Si-O-Ns	MK	Y_2_O_3_ (30–50 nm, I)	Eu_2_O_3_ (45-60 nm, C) CeO_2_ (20 nm, M)	Phosphor powders	[39]

Notes: Suppliers of nano-sized fillers: C = Cometox Srl, Milan, Italy; D = DGTech, Grenoble, France; E = Evonik Industries AG, Essen, Germany; G = Goodfellows, Huntingdon, U.K.; I = Inframat Advanced Materials, Manchester, CT; M = MKnano, M K Index Corp., Missisauga, Canada; P = PlasmaChem GmbH, Berlin, Germany; m-HAp: hydroxyalapatite micro-powders; b-HAp: hydroxyalapatite nano-powders; m-TiO_2_: titania micopowders; Polymers: MK, H44 and H62C (silicones) from Wacker Chemie AG, München, Germany; PSZ20: KiON Defence Technologies Inc., Huntingdon Valley, PA, USA;. NN120-20: Clariant AG, Sulzbach, Germany.

**Table 2.** Formulations used for the development of silicate coatings on Si-SiC foams.

Component	Amount used for Y-DS coating (wt%)	Amount used for zircon coating (wt%)
MK polymer	7	3
H62C polymer	–	5
Kaolin	1	2
Mullite powders	13	–
Zircon powders	–	12
Nano-Y_2_O_3_	12	–
Nano-ZrO_2_	–	11
Nano-TiO_2_	–	1
Isopropyl alcohol	67	66
